# Ammonia borane dehydrogenation and selective hydrogenation of functionalized nitroarene over a porous nickel–cobalt bimetallic catalyst†

**DOI:** 10.1039/c9ra01551e

**Published:** 2019-05-09

**Authors:** Hui Miao, Kelong Ma, Huiru Zhu, Kun Yin, Ying Zhang, Yumin Cui

**Affiliations:** School of Chemistry and Materials Engineering, Fuyang Normal University, Anhui Provincial Key Laboratory for Degradation and Monitoring of Pollution of the Environment Fuyang 236037 China huimiao@mail.ahnu.edu.cn

## Abstract

The hydrolysis of ammonia borane is a promising strategy for hydrogen energy exploration and exploitation. The *in situ* produced hydrogen could be directly utilized in hydrogenation reactions. In this work, a bimetallic nickel–cobalt material with porous structure was developed through the pyrolysis of ZIF-67 incorporated with Ni ions. Through the introduction of Ni(NO_3_)_2_ as an etching agent, the ZIF-67 polyhedrons were transformed into hollow nanospheres, and further evolved into irregular nanosheets. The bimetallic NiCo phase was formed after pyrolysis in a nitrogen atmosphere at high temperature, with the decomposition and release of organic ligands as gaseous molecules under flowing nitrogen. The obtained bimetallic NiCo porous materials show superior catalytic performance towards hydrolytic dehydrogenation of ammonia borane, thereby nitrobenzene with reducible functional groups can be reduced with high selectivity to the corresponding aniline.

## Abstract 2

Porous nickel–cobalt bimetallic catalyst realizes selective hydrogenation of nitrobenzene with *in situ* produced hydrogen through hydrolysis of ammonia borane.

## Graphical abstract

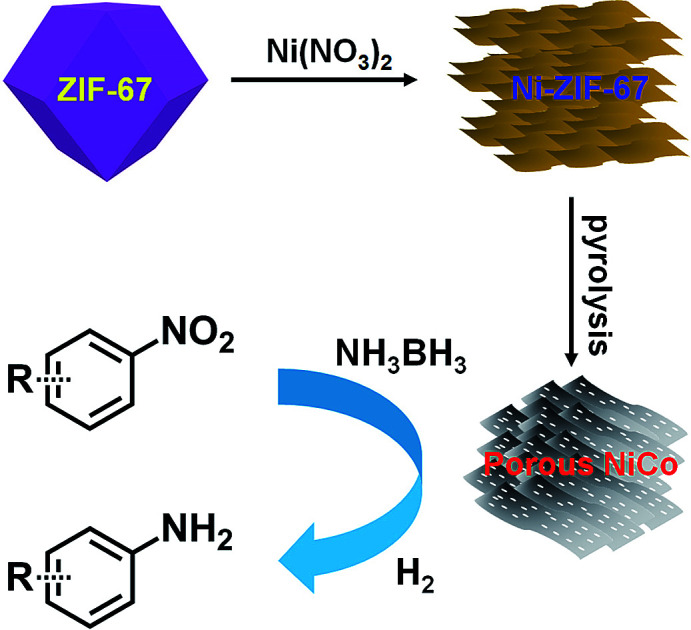

## 1. Introduction

Metal–organic frameworks (MOFs) are crystalline porous materials prepared by the self-assembly of metal ions and organic linkers, which have been used as templates or precursors for nanostructured porous materials.^1–4^ The morphology of MOF materials shows diversity because of the flexible and controllable composition, structure and pore size.^5^ They are attractive as ideal precursors for the preparation of porous nano-materials with various morphologies or structures.^6^ MOF-derived metal composites often show large surface area and hierarchical pore structures. Metal nanocrystals,^7^ oxides,^8^ sulfides,^9^ nitrides^10^ and phosphides^11^ have been successfully prepared through the evolution of MOFs, and have shown extraordinary performance in catalysis, sensing, energy storage and conversion, separation and other fields.^12^ ZIF-67 is a kind of zeolite imidazole skeleton material with Co^2+^ ions as metal sites and 2-methylimidazole as organic ligands.^13^ Through pyrolysis at high temperature, ZIF-67 converted into nitrogen doped carbon encapsulated Co/CoO_*x*_ nanocrystals (denoted as Co–N–C). The Co–N–C species has shown superior catalytic performance in both oxidation and reduction reactions.^14–16^ Through further phosphorization or sulfuration, the corresponding CoP–N–C or CoS–N–C can be used as an electrocatalytic material for the oxygen reduction reaction (ORR) or water splitting.^9,17^ The Co-based materials derived from might be promising alternative non-noble metal catalyst in heterogeneous catalysis.

Functional anilines are widely existed as key intermediates in dyes, pigments, herbicides and pharmaceuticals.^18^ The catalytic hydrogenation of nitroarenes using hydrogen as a reduce agent is the main protocol for the high efficiency and atom economic.^19^ Noble metal catalysts such as Pd and Pt have shown excellent catalytic activity in nitrobenzene hydrogenation.^20–23^ However, precious metals are rare, expensive and difficult to control the selectivity of hydrogenation. It is imperative to find non-precious metal catalysts that can replace precious metals. It has been reported that non-noble metal catalysts such as Fe and Co have been used in nitrobenzene hydrogenation.^24–26^ Actually, the direct use of hydrogen is uneconomical and increases the risk of reaction. Catalytic hydrogenation of non-noble metals generally requires higher reaction temperature and pressure.^27–30^ It is highly desirable to realize selective hydrogenation of nitrobenzene over non-noble metal catalysts under mild conditions.

Ammonia borane (NH_3_BH_3_, AB) is non-toxic, safe, stable and environmentally friendly compounds with high quality hydrogen storage (mass fraction of 19.6%).^31,32^ Based on these characteristics, it is the most potential new type of hydrogen storage.^33–35^ To combine the dehydrogenation of ammonia borane and hydrogenation of nitrobenzene would be an ideal strategy to realize selective hydrogenation of nitrobenzene with reducible functional groups under mild condition.^36^ To find cheap and efficient catalysts to reduce the cost of hydrogen production would be highly desirable but challenging. The non-precious metal catalysts, nickel and cobalt based nanoparticles or alloy catalysts have shown superior catalytic effect in hydrogen production by ammonia borane hydrolysis.^37,38^

The convenient and effective preparation strategy of non-noble catalysts is still a challenge due to the lower electronegativity.^39^ Thermal annealing with high temperature is always need, which often results in serious agglomeration and losing of surface area or active sites. To construct porous structure materials is propitious to expose more active site and mass transfer of substances in catalysis.^40^ The developed porous catalysts such as Ni, Au, Cu, *etc.* have shown improved or enhanced performance in many catalytic processes.^41–43^ In general, the synthetic methods of porous catalysts are based on template, corrosion or assembling of smaller particles.^44,45^ To develop novel, more convenient and effective synthetic method is crucial to higher requirements in catalysis. Here, we present a convenient and effective method to synthesize porous NiCo bimetallic catalyst with controllable composition through pyrolysis of Ni-ZIF-67. The obtained NiCo bimetallic catalysts show excellent activity towards hydrogen production by hydrolysis of ammonia borane.

## 2. Experimental

### 2.1 Materials

Co(NO_3_)_2_·6H_2_O (99%) and Ni(NO_3_)_2_·6H_2_O (99%) were purchased from Aladdin. Ammonia borane (90%) was purchased from Aldrich. Methanol and 2-methylimidazole were of analytical grade from the Sinopharm Chemical Reagent. All kinds of nitrobenzenes were of analytical grade from the Energy Chemical. All the reagents in this work were used without further purification.

### 2.2 Synthesis of ZIF-67

In a typical synthesis, 0.5 g Co(NO_3_)_2_ was dissolved in 15 mL of methanol; then 0.5 g 2-methylimidazole was dissolved in 15 mL of methanol. Then both solutions were mixed and stirred for 24 h at room temperature, then the resulting ZIF-67 solids were separated by centrifuging and washed with methanol for 3 times, and finally dried at 50 °C at vacuum for 24 h.

### 2.3 Synthesis of Ni-ZIF-67

The obtained ZIF-67 (0.1 g) was dispersed in 30 mL methanol and 0.1 g Ni(NO_3_)_2_ with 10 mL methanol was introduced, then stirred for 24 h at room temperature. The Ni-ZIF-67 was obtained after centrifuging and washing with methanol for 3 times. The resulting Ni-ZIF-67 solids were finally dried at 50 °C at vacuum for 24 h.

### 2.4 Synthesis of bimetallic NiCo porous materials

100 mg of Ni-ZIF-67 solid was placed into a quartz boat, which was charged into the middle of a quartz tube in a furnace with a continuous nitrogen flow of 50 mL min^−1^. The furnace was heated from room temperature to the targeted temperature (600–900 °C) with a programmed heating rate of 2.5 °C min^−1^. After the pyrolysis for 2 hours, the sample was naturally cooled down to room temperature. The resultant samples were denoted as NiCo-*T* (*T* denotes as pyrolysis temperature).

### 2.5 Characterization

X-ray diffraction (XRD) was performed on a Philips X'pert diffractometer equipped with a Ni-filtered Cu Kα radiation source. The particle size and morphology of as-synthesized samples were determined by using Hitachi model H-800 transmission electron microscope. High-resolution transmission electron microscopy (HRTEM) images were taken using a JEM-2100 electron microscope. X-ray photoelectron spectroscopy (XPS) experiments were performed on a ULVAC PHI Quantera microprobe. Binding energies (BE) were calibrated by setting the measured binding energies of C 1s to 284.6 eV.

### 2.6 Dehydrogenation of ammonia borane

The catalysts were suspended in water (5.0 mL) of a two-necked round-bottomed flask. A gas cylinder filled with water was connected to one neck of the reaction flask (the other neck was sealed) to measure the volume of hydrogen. The reaction was initiated in a water bath at 25 °C under ambient atmosphere. 1 mmol of ammonia borane was added into the reaction flask with stirring. By measuring the displaced water, the produced volume of hydrogen could be recorded.

### 2.7 Hydrogenation of nitrobenzene

In a typical reaction, nitrobenzene (0.5 mmol) was charged into a round bottom flask with ethanol (0.5 mL) and deionized water (5 mL). Then porous NiCo bimetallic catalyst (0.02 mmol) was introduced. After ammonia borane (1 mmol) was added, the round bottom flask was sealed immediately and the reaction mixture was stirred at 25 °C. The conversion and selectivity were determined by GC using *n*-hexadecane (100 μL) internal standard.

## 3. Results and discussion

### 3.1 Characterization of bimetallic NiCo porous materials

**Scheme 1:**
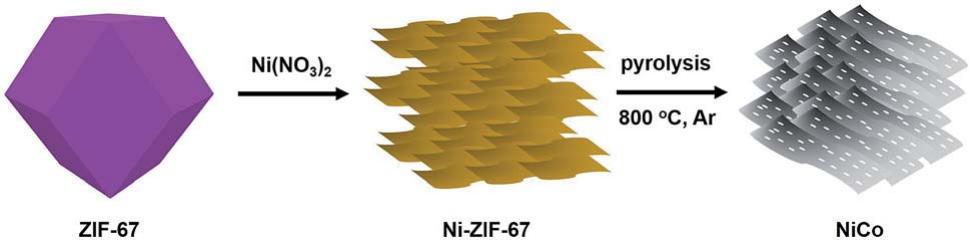
Schematic illustration of the synthetic route to porous NiCo bimetallic catalyst.

The synthetic route of NiCo bimetallic catalyst is schematically shown in Scheme 1. The corresponding structure model for the ZIF-67-Co rhombic dodecahedron is schematically illustrated. ZIF-67 was chosen for its high content of cobalt, which could be promising precursor for the synthesis of Co nanoparticles or metal oxide. Ni(NO_3_)_2_ was introduced as etching agents to transform the ZIF-67 polyhedrons into hollow nanospheres, and irregular nanosheets were subsequently generated with further increase the Ni salt. After pyrolysis in nitrogen atmosphere at high temperature, the Ni^2+^ and Co^2+^ were reduced *in situ* into bimetallic NiCo phase. Meanwhile, the organic ligand decomposed into gaseous molecule and released at high temperature under flowing nitrogen.

**Fig. 1:**
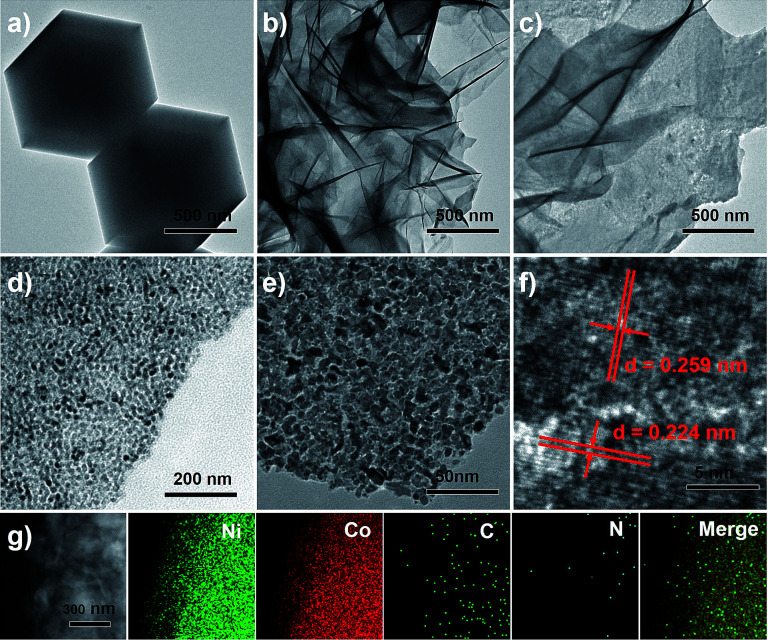
(a) TEM image of representative ZIF-67 nanocrystals; (b) TEM image of Ni-ZIF-67 with Ni/Co ratio 1 : 1; (c) TEM image of NiCo bimetallic materials obtained from pyrolysis of Ni-ZIF-67 at 800 °C; (d and e) the magnified TEM images of NiCo bimetallic materials; (f) HRTEM image of NiCo bimetallic materials; (g) EDS-mapping of NiCo bimetallic materials.

Through the characterization by Transmission Electron Microscopy (TEM), ZIF-67 with polyhedrons shape and smooth surface which have a diameter of 700 nm and an edge length of about 500 mm could be obtained according to reported methods (Fig. 1a).^15^Fig. 1a shows that the architectures are constructed from many wrinkled nanosheets. Based on the incremental amount of Ni salt, ZIF-67 polyhedrons were firstly etched into hollow nanospheres and then destroyed by more Ni salt into nanosheets (Fig. S1†). After pyrolysis of the eventually obtained Ni-ZIF-67 at 800 °C, the Ni^2+^ and Co^2+^ were simultaneously reduced into NiCo crystalline, which exhibit the synthesized architectures are composed of sheet-like nanostructures. Interestingly, different from the reported work about the organic ligand of ZIF-67 convert into nitrogen doped carbon materials, we found that no carbon was formed and only metallic NiCo was produced. The magnified TEM image (Fig. 1b) confirms the nanoporous structures of these nanosheets. The NiCo bimetallic material displays the honeycomb-like microstructures with pores, in which small NiCo nanoparticles interconnect with each other, forming a large number of pores, and interstitial structures on their exterior surfaces. Based on the characterization results, it is reasonably supported that the pyrolysis of the Ni-ZIF-67 would induce thermal decomposition of organic ligands and the release of a mass of gaseous molecule from the interior of the nanosheets, thus leading to the formation of porosity left inside the particles. The High Resolution Transmission Electron Microscopy (HRTEM) image (Fig. 1f) shows the lattice fringes with interfringe distances of 0.259 nm and 0.224 nm, which is between the characteristics of Ni and Co crystal phases in the (200) plane and (220) plane respectively. The energy-dispersive X-ray spectroscopy (EDS) mapping profile provides evidence of a homogeneous distribution of nickel and cobalt (Fig. 1g) in bimetallic NiCo porous material. Almost no carbon elements and nitrogen elements can be detected, which is consistence with the previous inference about the disappearance of organic moiety. The above characterization on the NiCo architecture reveals that pores in the architecture are actually constructed by the highly packed NiCo nanoparticles.

**Fig. 2:**
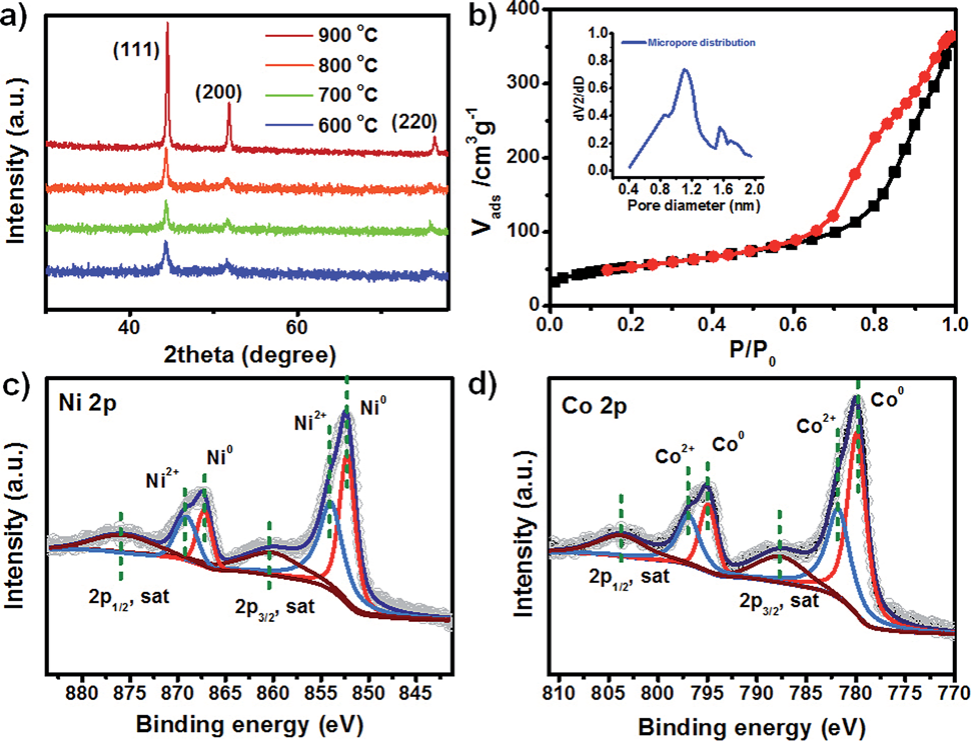
(a) The XRD patterns of the samples resulting from annealing Ni-ZIF-67 at various temperatures; (b) N_2_ adsorption/desorption isotherms and pore distribution of porous NiCo bimetallic catalyst obtained at 800 °C; XPS spectra of Ni 2p peaks (c) and Co 2p peaks (d) from Ni_1_Co_1_ bimetallic porous materials annealing at 800 °C.

To further study the conversion of ZIF-67 to Ni-ZIF-67 after adding Ni(NO_3_)_2_, the phase structure of the samples was analyzed by X-ray diffraction (XRD). The X-ray diffraction (XRD) patterns confirmed high crystallinity and zeolite type structure of ZIF-67 (Fig. S1†), and the diffraction peaks are identical to its corresponding simulated pattern. After the introduction of Ni salt, the XRD pattern of product changed notably with gradually increasing the amount (Fig. S2†). The characteristic peaks of ZIF-67 disappears, and the appearance of a new narrow diffraction peaks at 10° was found. After calcined at 800 °C, all of the detectable diffraction peaks agree well with standard data of cubic phase NiCo, and no other phases can be detected, indicating that the as-obtained calcined product is pure crystalline (Fig. 2a). Moreover, with increasing calcination temperature, the shape of these diffraction peaks become sharper and narrower. It is confirmed that the successful conversion of Ni-ZIF-67 to cubic phase NiCo after thermal treatment based on above HRTEM and XRD results. To evaluate the specific surface areas and porosity of the obtained porous NiCo bimetallic catalysts, the N_2_ adsorption–desorption isotherms and pore sizes were shown in Fig. 2b. The BET surface areas is 184.2 m^2^ g^−1^, and micropore size centered at 1.2 nm.

The chemical and electronic states of the NiCo architecture were further determined by X-ray photoelectron spectroscopy (XPS). All of the binding energies in the XPS analysis were corrected for specimen charging by referencing them to the C 1s peak (284.6 eV). Fig. 2c shows the high-resolution Ni 2p spectrum. It presents two prominent peaks at 852.4 and 867.5 eV, assigned to the Ni 2p_3/2_ and Ni 2p_1/2_ peaks respectively. Further deconvolution of the Ni 2p spectra yields two fitting peaks at binding energies of 852.2 and 867.2 eV are ascribed to Ni^0^, while another two fitting peaks at 854.0 and 869.2 eV are attributed to Ni^2+^. Fig. 2d shows the deconvolution of high-resolution Co 2p spectrum, and the Co 2p spectra yields two fitting peaks at binding energies of 779.9 and 794.9 eV are ascribed to Co^0^, while another two fitting peaks at 781.9 and 796.9 eV are attributed to Co^2+^. Therefore, the main valence state of NiCo bimetallic porous materials is zero with partial oxidation on the surface when exposing to air.

### 3.2 Catalytic activity in dehydrogenation of ammonia borane

**Fig. 3:**
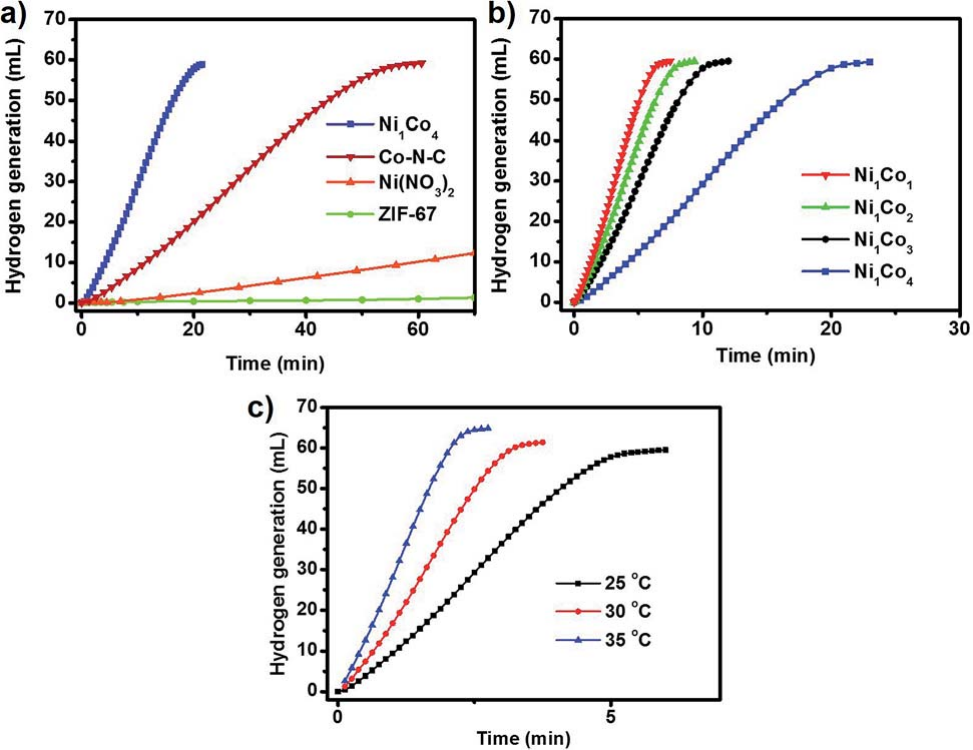
(a) Volume of hydrogen evolved *versus* reaction time for AB dehydrogenation reaction (1 mmol) catalyzed by the different catalysts; (b) volume of hydrogen evolved *versus* reaction time for AB dehydrogenation reaction catalyzed by the as-prepared bimetallic NiCo porous catalysts with different Ni/Co ratio; (c) volume of hydrogen evolved *versus* reaction time for AB dehydrogenation reaction catalyzed by the as-prepared bimetallic Ni_1_Co_1_ porous catalysts with different temperatures.

The as-prepared porous NiCo bimetallic catalysts were tested for the hydrolytic dehydrogenation of AB. For the Ni_1_Co_4_ catalysts, a rapid (20 minutes) and nearly linear hydrogen evolution curves was obtained without observable induction period (Fig. 3a). As the catalyst obtained from pyrolysis of pure ZIF-67 (denoted as Co–N–C, Fig. S3 and S4†) was employed in the hydrogen generation of aqueous AB, moderate activities for the hydrolysis was found (60 minutes). The precursors were also studied as catalyst for the hydrolytic dehydrogenation of AB. The pure Ni(NO_3_)_2_ and ZIF-67 show little activity and negligible activities respectively. The difference in the activity may be ascribed to the introduction of Ni in Co–N–C structure and the electronic effects between Ni and Co. Further reasonable modification of the metal ratios showed that the reaction time decreased with the increasing of Ni amount, and the Ni_1_Co_1_ could complete the reaction during just 7 minutes (Fig. 3b). The temperature dependence of the catalytic reaction was studied in the range 25–35 °C, with initial AB and Ni_1_Co_1_ concentrations of 1 and 0.05 mmol, respectively; the results are shown in Fig. 3c. When the hydrogen generation of aqueous AB was initiated at 30 °C and 35 °C, the reaction could be completed at three and two minutes.

### 3.3 Catalytic activity in hydrogenation of nitrobenzene

To demonstrate the general applicability of nitroarenes reduction of the porous NiCo bimetallic catalyst, we explored the hydrogenation of various nitroarenes with reducible functional groups through AB dehydrogenation (Table 1). Cyano group substituted nitrobenzene at *para*-position converted into aniline by nearly 100% conversion and selectivity (entry 1). The reduction of ester or amide substituted nitrobenzene produced corresponding anilines with 88% or 85% conversion (entries 2 and 3). Particularly, the most-challenging nitrobenzene with ethynyl or vinyl substituted could also be reduced into ethynyl or vinyl aniline with 97% or 99% conversion and near 100% selectivity (entries 4 and 5). Halogenated nitrobenzenes, such as chloro-, bromo-substituted, were converted into halogenated anilines in excellent conversion (89–95%) and selectivity (95–98%) (entries 6 and 7). Moreover, 3-nitropyridine compound could also be reduced into the 3-aminopyridine which is an important building blocks in pharmaceuticals and agrochemicals with 98% conversion and 100% selectivity (entry 8). The above results show that the porous NiCo bimetallic catalyst is highly effective in AB dehydrogenation and chemo-selective hydrogenation of functionalized nitrobenzenes. Compared with the reported catalysts (Table S1†), our catalysts can achieve the mildest reaction conditions for nitrobenzene hydrogenation, and comparable catalytic activity towards combination of ammonia borane dehydrogenation and nitrobenzene hydrogenation.

**Table 1:** The hydrogenation of functionalized nitrobenzene through ammonia borane dehydrogenation with porous NiCo bimetallic catalyst

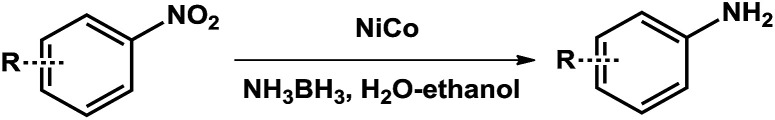				
Entry^a^	Substrate	Product	Con.^b^ (%)	Sel.^b^ (%)
1	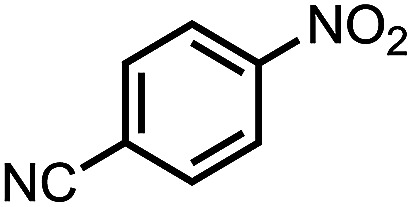	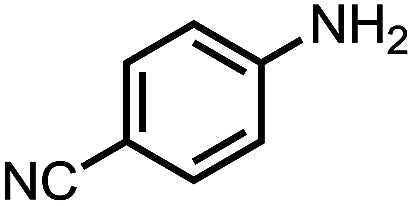	99	100
2	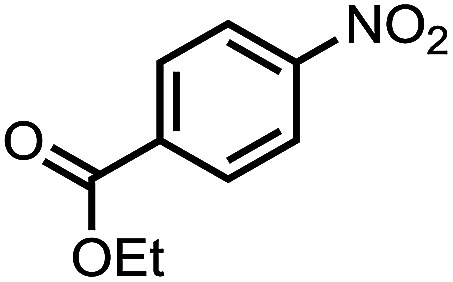	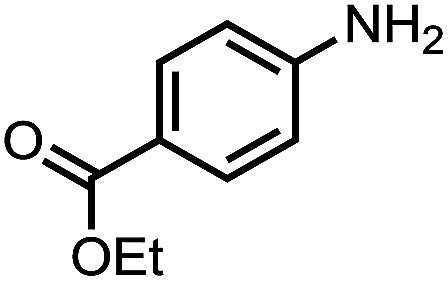	88	100
3	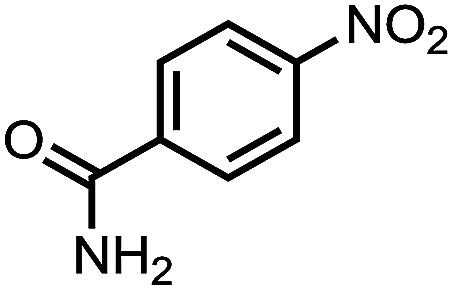	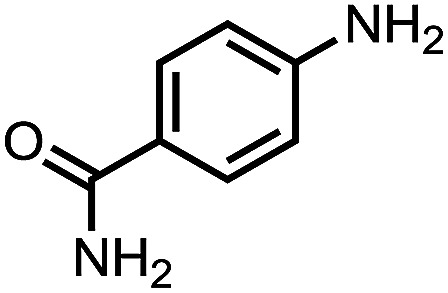	85	100
4	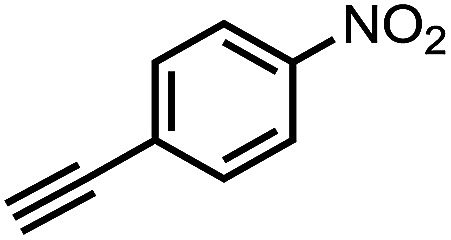	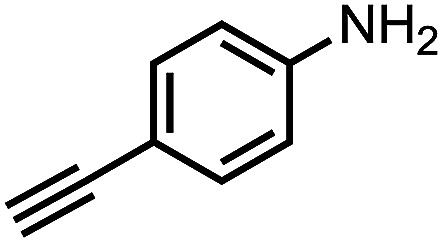	97	100
5	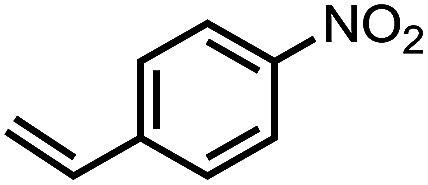	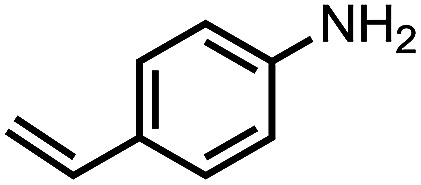	99	99
6	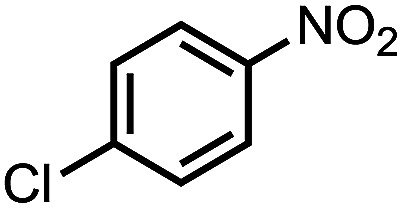	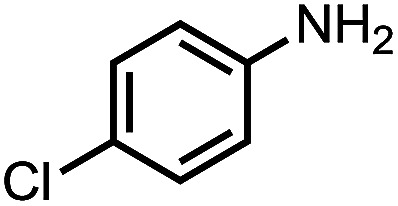	89	95
7	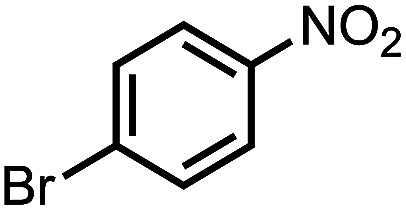	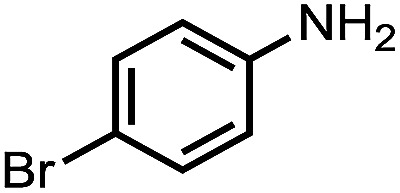	95	98
8	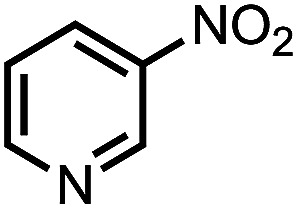	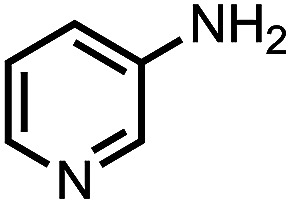	98	100

a Reaction condition: 0.5 mmol nitroarene, 0.02 mmol catalyst, ammonia borane (1 mmol), 25 °C, 2 hours, 10 : 1 water–ethanol (5.5 mL).

b Conversion and selectivity were determined by GC using *n*-hexadecane (100 μL) internal standard.

### 3.4 Recycle and stability of bimetallic NiCo porous materials

**Fig. 4:**
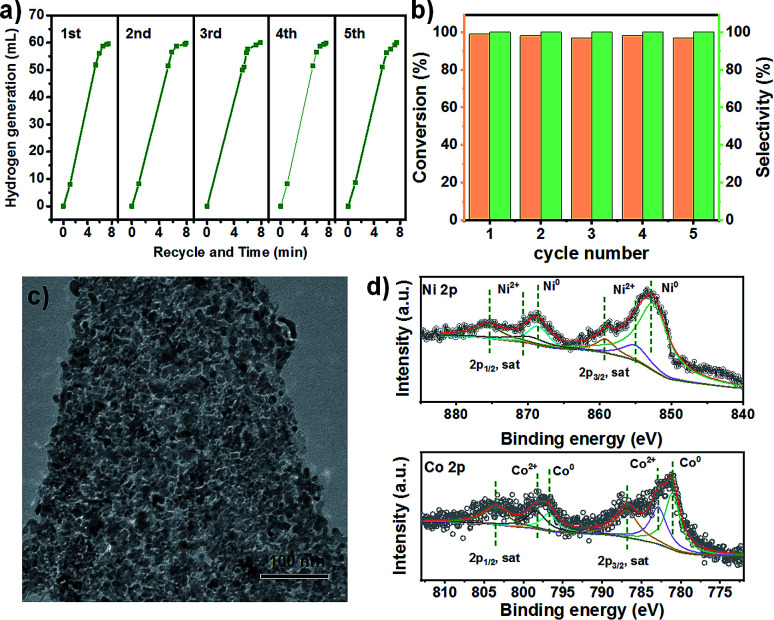
(a) The cycle experiments of ammonia borane dehydrogenation over porous NiCo bimetallic catalyst; (b) the cycle results of nitrobenzene hydrogenation over porous NiCo bimetallic catalyst (using 4-nitrobenzonitrile as substrate); (c) the TEM image of porous NiCo bimetallic catalyst after five cycles; (d) the XPS spectra of porous NiCo bimetallic catalyst after five cycles.

The bimetallic NiCo porous materials used in nitrobenzene hydrogenation could be easily recovered by an applied magnetic field after the reaction. The test of AB dehydrogenation over bimetallic NiCo porous materials was repeated five times to evaluate the catalyst stability. After each experiment, the catalyst was separated from the reaction products by an applied magnetic field, washed with water and reused in the next cycle. As shown in Fig. 4a, which indicates that the bimetallic NiCo porous materials remain stable under the reaction conditions. Bimetallic NiCo porous materials can remain effective activity and selectivity after five cycle reactions. Moreover, we also tested the cycle performance of bimetallic NiCo porous materials in the hydrogenation of nitrobenzene with AB dehydrogenation. The results showed that there was no significant decline in catalytic activity of bimetallic NiCo porous materials after five cycles (Fig. 4b). The spent catalysts were characterized after five cycles and found that NiCo still maintained the morphology as the initial catalyst (Fig. 4c), and the surface state of the catalysts did not change significantly (Fig. 4d). These results reveal that mild reaction conditions are not enough to change the overall structure of the catalyst, and the reductive reaction conditions avoid the oxidation of NiCo in air. The mild reaction condition, ultra-high catalytic efficiency and stability would be advantageous and promising in the practical application.

## 4. Conclusions

In conclusion, the convenient and effective preparation strategy for the bimetallic NiCo porous materials has been developed. Through the introduction of Ni(NO_3_)_2_ as etching agents, the ZIF-67 polyhedrons were transformed into hollow nanospheres, and irregular nanosheets with further increasing the amount of Ni salt. The bimetallic NiCo phase was formed after pyrolysis in nitrogen atmosphere at high temperature, with the decomposition and releasing of organic ligand into gaseous molecule under flowing nitrogen. The as-synthesized bimetallic NiCo porous materials show superior activity towards the hydrolytic dehydrogenation of AB. The application of AB dehydrogenation in hydrogenation reaction has perfectly realized the chemo-selective reduction of nitrobenzene with reducible functional groups. The etching through metal salt and subsequent pyrolysis at high temperature is a novel and promising strategy for the construction of porous metal materials in catalysis.

## Conflicts of interest

There are no conflicts to declare.

## Supplementary Material

RA-009-C9RA01551E-s001
