# Supplementary material for: Ammonia borane dehydrogenation and selective hydrogenation of functionalized nitroarene over a porous nickel–cobalt bimetallic catalyst
Source: RSC Adv. 2019 May 9;9(26):14580–5. doi: 10.1039/c9ra01551e (PMC9064148; doi:10.1039/c9ra01551e)
Supplement: RA-009-C9RA01551E-s001 [file RA-009-C9RA01551E-s001.pdf]

Electronic Supplementary Material (ESI) for RSC Advances.
This journal is © The Royal Society of Chemistry 2019

Supporting Information

Ammonia Borane Dehydrogenation and Selective Hydrogenation of Functionalized Nitroarene over Porous Nickel-Cobalt Bimetallic Catalyst

Hui Miao,*[a] Kelong Ma, [a] Huiru Zhu, [a] Kun Yin, [a] Ying Zhang [a] and Yumin Cui [a]

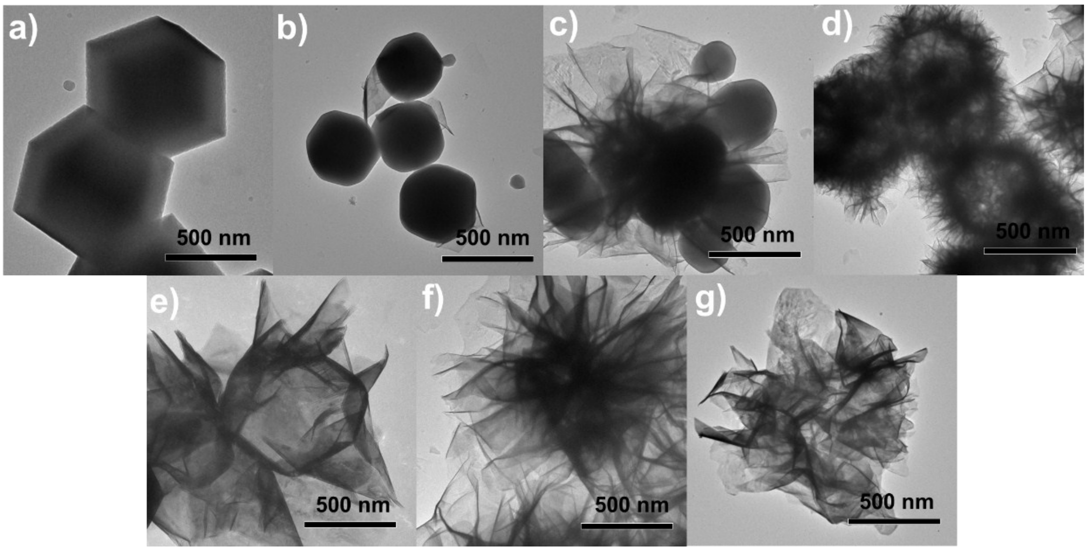

Figure S1. TEM images of Ni-ZIF-67 with different Ni:Co ratio, a) 1:8, b) 1:7, c) 1:6, d) 1:5, e) 1:4, f) 1:3, g) 1:2.

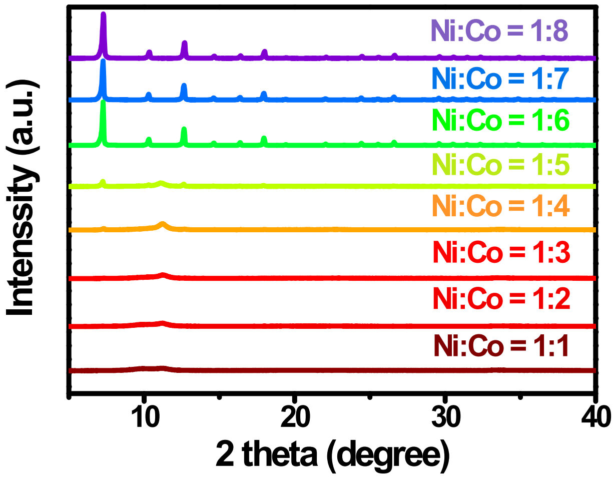

Figure S2. The XRD patterns of the samples resulting from introduction of $Ni(NO_3)_2$ to ZIF-67 with gradually increasing amount.

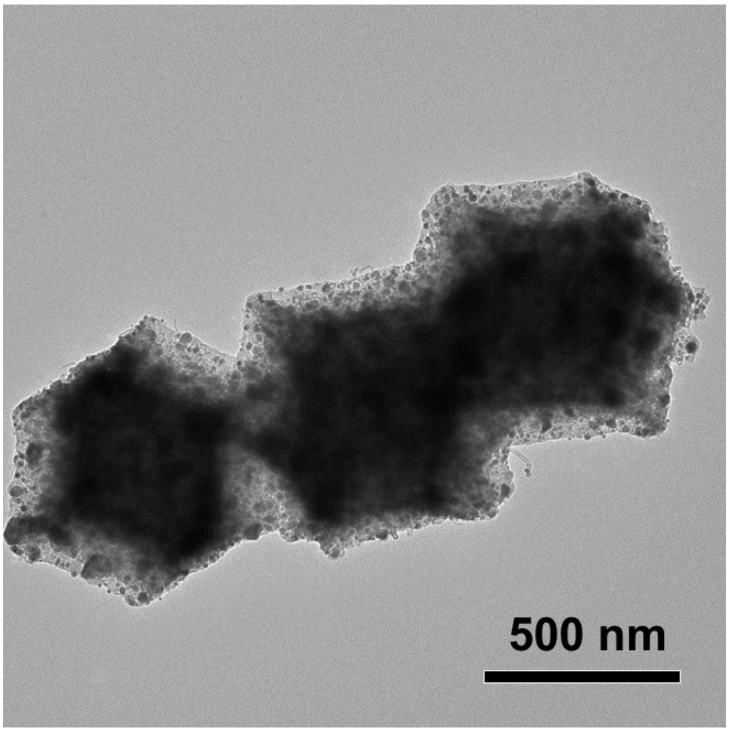

Figure S3. TEM image of Co-N-C derived from pyrolysis of ZIF-67 at 800 ^{o}C under nitrogen atmosphere.

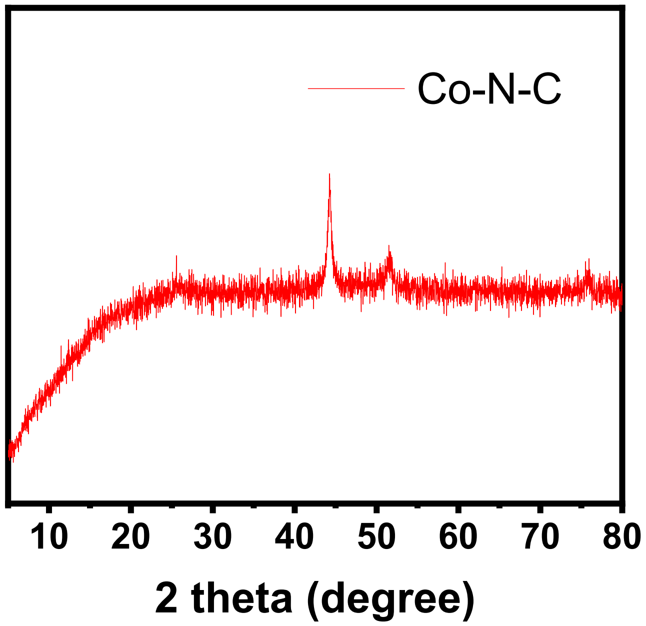

Figure S4. XRD patterns of Co-N-C derived from pyrolysis of ZIF-67 at 800 ^{o}C under nitrogen atmosphere.

Table S1. The comparision of bimetallic NiCo porous catalyst with other reported catalysts in nitrobenzene hydrogenation reaction.

Entry	Catalyst	Hydrogen source	Conditions	Yields	Ref.
**1**	**NiCo (ZIF-67)**	**AB dehydrogenation**	**2 h, 25 ^{o}C,**	**100**	**Our work**
2	Co/CoO (ZIF-67)	AB dehydrogenation	1.5 h, 25 ^{o}C, MeOH, H_2O	100	Chem. Commun., 2016, 52, 7719
3	Co_3S_4 (ZIF-67)	Na_2S, Na_2SO_3 + H_2O	2.5 h, 25 ^{o}C, H_2O	99.8	Catal. Commun. 2017, 101, 31
4	Single atom Co (ZIF-8/ZIF-67)	H_2	4.5 h, 110 ^{o}C, 3 MPa	100	Journal of Catalysis 357 (2018) 20
5	Co nanoparticles (ZIF-67)	H_2	3 h, 100 ^{o}C, 1 MPa	99	J. Mol. Catal. A-Chem. 2016, 420, 56
6	Co_2P (ZIF-67)	H_2	6 h, 60 ^{o}C, 5 MPa	99	Chem. Eur. J. 2018, 24, 4234
7	Co@Pd/CN (ZIF-67)	H_2	45 min, 25 ^{o}C, 1atm	99	ACS Catal. 2015, 5, 5264
8	Co@C-N (ZIF-67)	Isopropanol	80 h, 80 ^{o}C	80	Chem. Commun., 2015, 51, 2331
9	γ-Fe_2O_3 (Fe-MIL-88A.)	$N_2H_4{\cdot}H_2O$	1 h, 85 ^{o}C	100	Chem. Commun., 2016, 52, 4199
10	Fe/Fe_2O_3 (Fe-MIL-101)	$N_2H_4{\cdot}H_2O$	0.3h, 40 ^{o}C	100	ChemCatChem 2019, 11, 724
